# Altered microRNA expression and pre-mRNA splicing events reveal new mechanisms associated with early stage *Mycobacterium avium* subspecies *paratuberculosis* infection

**DOI:** 10.1038/srep24964

**Published:** 2016-04-22

**Authors:** Guanxiang Liang, Nilusha Malmuthuge, Yongjuan Guan, Yuwei Ren, Philip J. Griebel, Le Luo Guan

**Affiliations:** 1 Department of Agricultural, Food and Nutritional Science, University of Alberta, Edmonton, AB, Canada; 2 UWA Institute of Agriculture and School of Animal Biology, University of Western Australia, Crawley, WA, Australia; 3 Department of Animal Biology, School of Veterinary Medicine, University of Pennsylvania, Philadelphia, Pennsylvania, United States of America; 4 Key Laboratory of Agricultural Animal Genetics, Breeding and Reproduction of Ministry of Education, Huazhong Agriculture University, Wuhan, Hubei, China; 5 Vaccine and Infectious Disease Organization, University of Saskatchewan, Saskatoon, SK, Canada; 6 School of Public Health, University of Saskatchewan, Saskatoon, SK, Canada

## Abstract

The molecular regulatory mechanisms of host responses to *Mycobacterium avium* subsp. *paratuberculosis* (MAP) infection during the early subclinical stage are still not clear. In this study, surgically isolated ileal segments in newborn calves (n = 5) were used to establish *in vivo* MAP infection adjacent to an uninfected control intestinal compartment. RNA-Seq was used to profile the whole transcriptome (mRNAs) and the microRNAome (miRNAs) of ileal tissues collected at one-month post-infection. The most related function of the differentially expressed mRNAs between infected and uninfected tissues was “proliferation of endothelial cells”, indicating that MAP infection may lead to the over-proliferation of endothelial cells. In addition, 46.2% of detected mRNAs displayed alternative splicing events. The pre-mRNA of two genes related to macrophage maturation (monocyte to macrophage differentiation-associated) and lysosome function (adenosine deaminase) showed differential alternative splicing events, suggesting that specific changes in the pre-mRNA splicing sites may be a mechanism by which MAP escapes host immune responses. Moreover, 9 miRNAs were differentially expressed after MAP infection. The integrated analysis of microRNAome and transcriptome revealed that these miRNAs might regulate host responses to MAP infection, such as “proliferation of endothelial cells” (bta-miR-196 b), “bacteria recognition” (bta-miR-146 b), and “regulation of the inflammatory response” (bta-miR-146 b).

Johne’ s disease (JD) is chronic granulomatous enteritis of ruminants caused by *Mycobacterium avium* subspecies *paratuberculosis* (MAP)^1^. Clinical symptoms of JD in cattle include persistent diarrhea, progressive weight loss, decreased production and death^2^. Although only 10–15% of MAP-infected cattle may develop clinical disease^3^,^4^, the carriers shed MAP into feces and milk^5^, which are the main sources of infection for other animals and possibly a zoonotic threat to humans^6^. To date, vaccines for JD are capable of controlling MAP shedding and clinical disease, but are not effective in preventing MAP infection^7^. In addition, animals infected by MAP usually undergo a long asymptomatic period and the diagnosis of MAP infection during the early subclinical stage remains challenging^8^,^9^.

MAP infects the gastrointestinal tract primarily through the ileum or distal small intestine during the first few months after a calf is born^10^,^11^. Bacteria enter via M-cells overlying lymphoid follicles in the ileal Peyer’s patches (PPs), and establish a persistent infection in submucosal macrophages^1^. Many studies have focused on the mechanisms of MAP infection by characterizing host innate and adaptive immune responses *in vitro* (macrogphage cell line) and *in vivo* (ileal tissue) during the subclinical period, revealing a pronounced effect on immune cells, the systemic immune system, and the mucosal immune system^12^,^13^. Recently, gene expression changes in MAP-infected macrophages and whole blood of MAP-infected calves have been reported^14^,^15^. However, little is known about transcriptome alterations and the molecular mechanisms regulating the host response to MAP at the site of infection during the subclinical stage of disease.

A previous study reported the existence of MAP in ileal tissues and MAP-specific immune responses (such as interferon gamma responses) in calves one month after MAP infection, suggesting that a persistent infection was established within one month post-infection^16^. Moreover, the post-transcriptional regulation by microRNAs (miRNAs) and alternative splicing can play a role in host responses to pathogenic bacteria^17^,^18^. Thus, we hypothesized that the regulatory mechanisms of miRNAs and alternative splicing of pre-mRNAs may be associated with host responses during persistent MAP infection. This study used an *in vivo* model to localize MAP infection to the terminal small intestine and studied gene expression and post-transcriptional regulation (miRNA expression and pre-mRNA splicing) at the site of infection one-month post-infection. These transcriptional and post-transcriptional changes provide new insights into the mechanisms by which MAP effectively evades host immune responses and establishes a persistent infection.

## Results

### RNA-Seq profiling of MAP-infected and control compartments of ileum tissues

**Figure 1:**
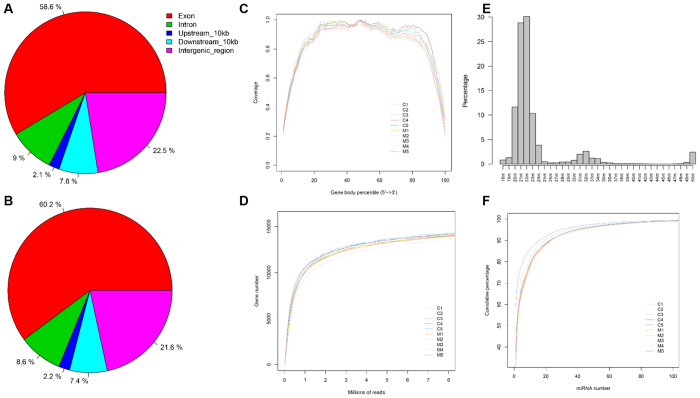
Quality control of RNA-Seq dataset. **(A)** The distribution of genomic locations of RNA-Seq reads in control compartment. **(B)** The distribution of genomic locations of RNA-Seq reads in infected compartment. **(C)** The plot of RNA-Seq coverage of gene body. **(D)** Saturation curve for gene number detection; X-axis - number of the mapped reads; Y-axis - number of the expressed genes (FPM> 1). **(E)** Length distribution of small RNA reads. **(F)** Cumulative frequency of detected miRNAs.

The surgically isolated ileum in calves (10–14 days old) was subdivided into two compartments; MAP-infected (infected) and non-infected (control). Intestinal tissues from each ileal compartment, including the PPs, were collected from all animals (n = 5) at one month post-infection (age = 40–44 days) and prepared for transcriptome profiling using RNA-Seq. A total of 300,676,396 paired-end sequence reads were obtained from the 10 samples. On average, ~89% of these reads were mapped to the bovine reference genome (UMD3.1, Supplementary Table S1). The quality of RNA-Seq data was evaluated via the genomic regions of reads, the RNA-Seq 3′/5′ bias and the sequencing depth. Approximately 78% of the reads were derived from exonic and intronic regions, gene upstream and downstream regions, whereas 22% were derived from intergenic regions (Fig. 1A,B). In addition, the coverage of reads along each transcript revealed no noticeable 3′/5′ bias, confirming the acceptable quality of sequencing data (Fig. 1C). The number of detected genes increased along with increasing sequencing reads and the gene number eventually plateaud, revealing that the most expressed genes were detected by RNA-Seq (Fig. 1D).

A total of 17,789,335 small RNA reads were obtained from the same 10 samples (Supplementary Table S1) and 58.94% of the total reads were 21 nt and 22 nt in length (Fig. 1E). The reads (15,207,875) with 18–25 nt lengths were used for further miRNA expression analysis. Among these reads, 14,062,510 reads were mapped to a known miRNA database (miRBase version 21), which resulted in 3,802 reads representing 55 putative novel miRNAs. The remaining 1,137,761 reads that were not identified as miRNAs belonged to other small noncoding RNAs (tRNA, snoRNA, snRNA and others). Cumulative frequency analysis revealed that the 20 most highly expressed miRNAs accounted for approximately 90% of the sequenced reads, representing the majority of expressed miRNAs (Fig. 1F).

### Transcriptome, alternative splicing events, and miRNA expression changes after MAP infection

**Figure 2:**
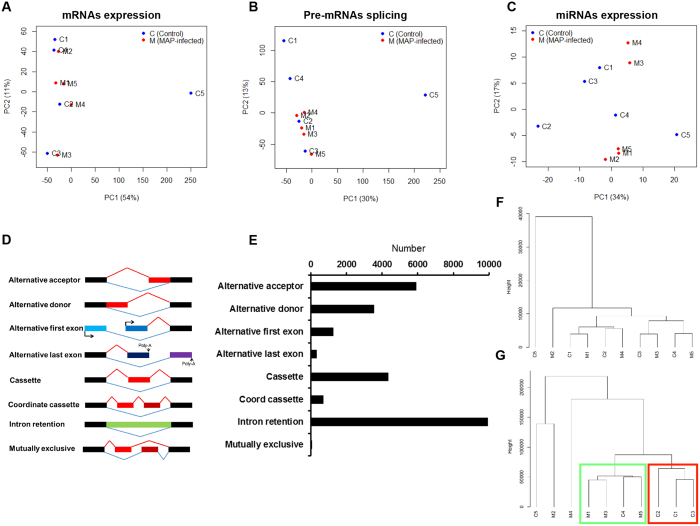
Profiling of mRNAs expression, alternative splicing events and miRNAs expression. **(A)** PCA plot of mRNAs expression. **(B)** PCA plot of alternative splicing events. **(C)** PCA plot of miRNAs expression. **(D)** Categories of alternative splicing events. **(E)** Number of different alternative splicing events. **(F)** Hierarchical cluster of mRNAs expression. **(G)** Hierarchical cluster of miRNAs expression. The red and green frames indicate two clusters based on miRNAs expression.

An average of 14,444 ± 187 (mean ± SD) and 14,430 ± 87 genes were identified (fragments per million mapped fragments (FPM) > 1) in control and infected ileal compartments, respectively. In total, 13,046 genes were commonly expressed in all 10 samples. Functions of the 3,000 most highly expressed genes were related to “metabolic process” and “protein synthesis” (Supplementary Table S2). Principal component analysis (PCA) and hierarchical cluster analysis showed no clear separation between infected and control samples (Fig. 2A,F). The transcriptome profile of animal #5 control compartment was an outlier from all control samples of other animals (Fig. 2A), thus this animal was subsequently removed from further analysis.

An average of 20,036 ± 3,163 alternative splicing events were detected in the control compartment tissues, whereas 21,005 ± 870 were detected in infected tissues. There were no significant differences between the two groups in terms of the number of alternative splicing events. However, the number of alternative splicing events differed greatly between control and infected compartments (14,598 *vs.* 19,661) of animal #5, which was also an outlier (Fig. 2B). These differences led us to exclude animal #5 from further pre-mRNA splicing analyses. The remaining four animals revealed 26,045 alternative splicing events (5,903 alternative acceptor, 3,535 alternative donor, 1,262 alternative first exon, 324 alternative last exon, 4,344 cassette, 705 coordinate cassette, 9,918 intron retention, and 54 mutually exclusive events) from 6,695 genes expressed in the ileal transcriptome (46.2% of total expressed genes) (Fig. 2D,E).

The number of detected miRNAs (reads per million mapped reads (RPM) > 1) was 375 ± 26 in infected compartments and 375 ± 25 in control compartments with 280 miRNAs commonly expressed in all 10 samples. The most highly expressed miRNA was miR-143. Although PCA did not show a clear separation (Fig. 2C), hierarchical cluster displayed a separation between the miRNA expression patterns of control and infected tissues (Fig. 2G). Unlike mRNA profile and alternative splicing events, the control compartment of animal #5 was not an outlier (Fig. 2C); however, to be consistent, this animal was excluded from further miRNAs differential expression analyses.

### Altered expression of mRNAs in MAP-infected ileal compartments

**Figure 3:**
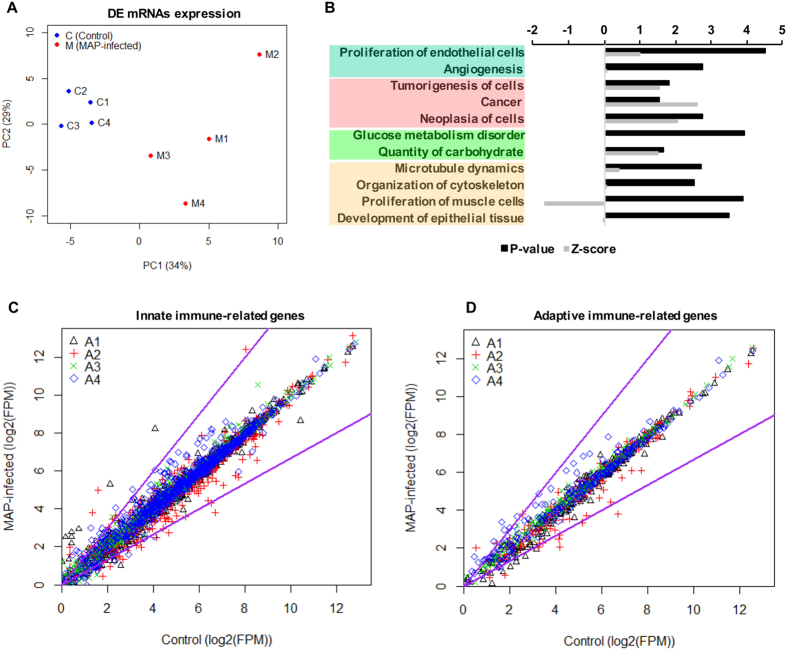
Analysis of differentially expressed mRNAs. **(A)** PCA plot of differentially expressed mRNAs. **(B)** Barplot of GO terms enrichment analysis. The X-axis represented the −log (*P* value), with longer bars indicating more relevant functions. **(C)** Fold change of innate immune-related genes when comparing control *vs*. infected tissues. Each dot denotes a single gene. The X-axis represents the log2 (FPM) value for each gene in control tissue, while Y-axis represents the log2 (FPM) value for each gene in infected tissues. Dots beyond the purple lines indicate fold change > 1.5. A1, A2, A3 and A4 represent Animal #1, Animal #2, Animal #3, Animal #4, respectively. **(D)** Fold change of adaptive immune-related genes when comparing infected *vs*. control tissues with data presented as described above. A1, A2, A3 and A4 represent Animal #1, Animal #2, Animal #3, Animal #4, respectively.

The analysis of differentially expressed (DE) mRNAs between infected (n = 4) and control compartments (n = 4) using edgeR^19^ revealed 81 DE genes (14 downregulated and 67 upregulated in infected tissues *vs*. control tissues; *P* < 0.05, fold change >1.5, Table 1). PCA plots revealed a clear clustering of these DE genes based on the ileal compartments (infected *vs*. control) (Fig. 3A). The most relevant function of DE genes estimated by the Ingenuity Pathway Analysis (IPA) was “proliferation of endothelial cells”, which showed an activated trend in infected tissues (z-score = 1.01) (Fig. 3B). Moreover, the IPA revealed that the DE genes were significantly related to “glucose metabolism disorder” and downregulated “proliferation of muscle cells” (z-score = −1.67) (Fig. 3B).

To evaluate the impact of MAP infection on host innate and adaptive immune responses, genes related to innate (793 ± 6, annotated by GO: 0045087) or adaptive immune responses (220 ± 3, annotated by GO: 0002250) were selected. Although some genes in certain individual animals revealed fold changes more than 1.5 (Fig. 3C,D), neither innate nor adaptive immune-related genes expressions showed statistical differences between two compartments (fold change >1.5, *P* < 0.05, paired t-test).

### Altered alternative splicing events of pre-mRNA in MAP-infected ileal compartments

**Figure 4:**
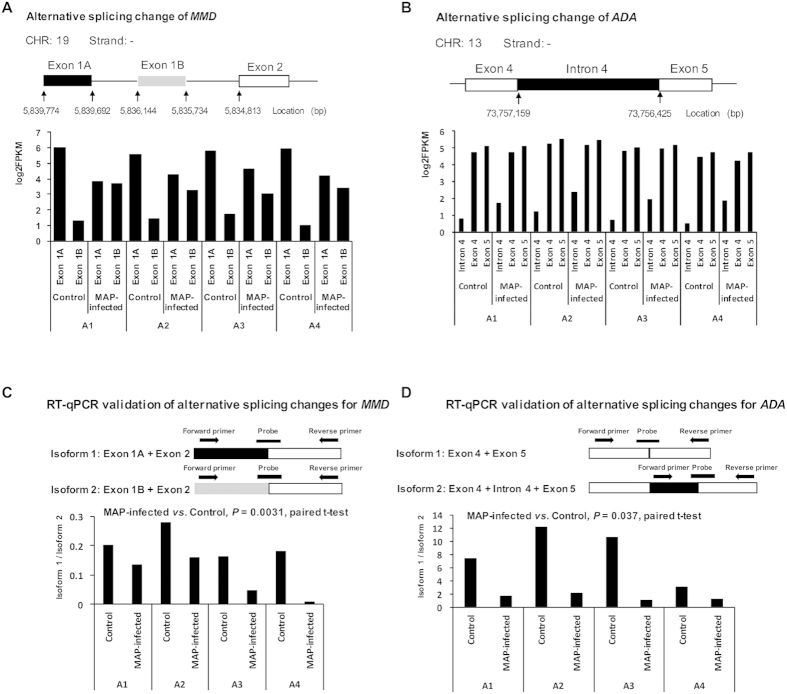
Analysis of alternative splicing events. **(A)** The alternative first exon event of *MMD*. The figure shows the genomic location of this event, and Exon 1A and Exon 1B were alternatively spliced as the first exon of *MMD* mRNA. The Y-axis represents the normalized expression level (log_2_FPKM) of Exon 1A or Exon 1B. **(B)** The intron retention event for *ADA*. The figure showed the genomic location of this event and Intron 4 was alternatively spliced. The Y-axis represented the normalized expression level (log_2_FPKM) of Exon 4, Exon 5 or Intron 4. RT-qPCR validation of alternative splicing changes for *MMD* **(C)** and *ADA* **(D)**. Primers and probes were designed based on each event. Y-axis of the bar plot represented the ratio between the expressions of two isoforms: isoform 1 / isoform 2. *significant difference at *P* < 0.05. A1, A2, A3 and A4 represent Animal #1, Animal #2, Animal #3, Animal #4, respectively.

To test the differences in alternative splicing events between control and infected compartments, Fisher’s exact tests were performed on raw read counts from 2 × 2 tables of exclusion and inclusion read counts for each animal as suggested by a previous study^20^. The differential alternative splicing events were identified in each animal (adjusted *P* < 0.05, Fisher’s exact test, Delta Percentage Splicing Index (ΔPSI) > 10%). There were 16 genes that displayed significantly different alternative splicing events between control and infected compartments of all four animals (Supplementary Table S3). Among them, an alternative first exon event was detected (adjusted *P* < 0.05, Fisher’s exact test, ΔPSI > 10%) in the mRNA of monocyte to macrophage differentiation-associated (*MMD*) (Fig. 4A). In MAP-infected tissues, the expression (fragments per kilobase of exon per million mapped reads (FPKM)) of exon 1A (the first annotated exon in bovine genome UMD 3.1) decreased among all 4 animals (log_2_FPKM in MAP-infected *vs*. log_2_FPKM in control: 6.0 *vs*. 3.8 in Animal #1; 5.6 *vs*. 4.3 in Animal #2; 5.8 *vs*. 4.6 in Animal #3; 6.0 *vs*. 4.2 in Animal #4) (Fig. 4A), while the expression of exon 1B (the alternatively spliced form for the first exon) increased in all animals (log_2_FPKM in MAP-infected *vs*. log_2_FPKM in control: 1.3 *vs*. 3.6 in Animal #1; 1.5 *vs*. 3.2 in Animal #2; 1.7 *vs*. 3.1 in Animal #3; 1.0 *vs*. 3.4 in Animal #4) when compared to control tissues (Fig. 4A). In addition, an intron retention event was detected (adjusted *P* < 0.05, Fisher’s exact test, ΔPSI > 10%) in adenosine deaminase (*ADA*) transcript (Fig. 4B). A higher expression of intron 4 (intron region between exon 4 and exon 5 of the *ADA* mRNA) were detected in MAP-infected tissues among all 4 animals, compared to that of control tissues (log_2_FPKM in MAP-infected *vs*. log_2_FPKM in control: 1.7 *vs*. 0.8 in Animal #1; 1.2 *vs*. 2.4 in Animal #2; 0.7 *vs*. 2.0 in Animal #3; 0.5 *vs*. 1.9 in Animal #4) (Fig. 4B). Subsequently, further protein sequence analysis (translate RNA sequence to protein sequence) on the above alternative spliced forms of *MMD* and *ADA* mRNAs in infected tissues revealed the potential introduction of stop codons, when the detected alternative splicing events happen (Supplementary Table S4). The multiplex reverse transcription quantitative PCR (RT-qPCR) was further performed to verify the differences between MAP-infected and control tissues in alternative spliced forms of above two genes. The primers and probes were designed to target the identical isoforms that were detected by RNA-Seq. Using the primers and probes designed; only two isoforms were detected for both genes (data not shown). The ratio between the expression of isoform 1 (exon 1A + exon 2) and isoform 2 (exon 1B + exon 2) for *MMD* decreased significantly (*P* = 0.0031, paired t-test) in infected tissues (0.21 ± 0.05) when compared to control tissues (0.10 ± 0.05) (Fig. 4C). Similarly, the ratio between the expression of isoform 1 (exon 4 + exon 5) and isoform 2 (exon 4 + intron 4 + exon 5) for *ADA* decreased significantly (*P* = 0.037, paired t-test) in infected tissues (8.26 ± 4.02) when compared to control tissues (1.48 ± 0.48) (Fig. 4D). RT-qPCR confirmed that the expression of isoform 2 for both genes was upregulated in the infected tissues *vs*. control tissues.

### Altered miRNAs expression in MAP-infected ileal compartments

**Figure 5:**
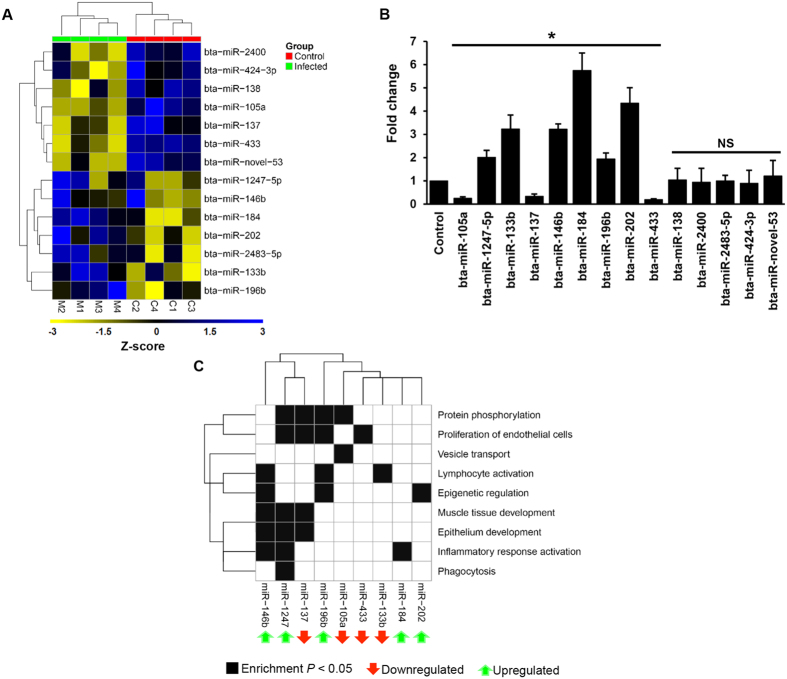
Analysis of differentially expressed miRNAs. **(A)** Heatmap of differentially expressed miRNAs based on RNA-Seq expression data. Blue indicates a higher expression level, whereas yellow represents a lower expression level. **(B)** Validation of miRNA expression using stem-loop RT-qPCR. Y-axis presents fold change of miRNA when infected tissues were compared to control tissues. *significant difference at *P* < 0.05; NS: no significant difference. Data presented as mean ± standard error of the mean (SEM). **(C)** GO term enrichment of miRNA targeted genes. Targeted genes were identified on the basis of two criteria: computational prediction and negative correlation analysis, r < −0.5, *P* < 0.05, Pearson’s correlation analysis. Black squares represent functions with significant enrichment (*P* < 0.05) that were identified by PANTHER. Red/green arrows indicate miRNAs were down-/up-regulated, respectively, in infected compartments.

A total of 14 DE miRNAs were identified when comparing infected (n = 4) and control (n = 4) compartments of the ileum (*P* < 0.05, edgeR paired group comparison, fold change >1.5). The expression of bta-miR-105a, bta-novel-53, bta-miR-433, bta-miR-2400, bta-miR-137, bta-miR-424–3p, and bta-miR-138 was downregulated in the infected compartment, when compared to the control (Fig. 5A). In contrast, the expression of bta-miR-146 b, bta-miR-196 b, bta-miR-2483–5p, bta-miR-133b, bta-miR-1247-5p, bta-miR-184, and bta-miR-202 was upregulated in the infected compartment, when compared to the control (Fig. 5A). Stem-loop RT-qPCR results confirmed differential expression patterns of 9 out of 14 of these miRNAs (bta-miR-105a, bta-miR-133b, bta-miR-137, bta-miR-146 b, bta-miR-184, bta-miR-196 b, bta-miR-202, bta-miR-433, and bta-miR-1247-5p) revealed by RNA-Seq (Fig. 5B).

### Integrated analysis of miRNA and mRNA networks

The integrated analysis of miRNA and mRNAs was performed using a two-step method. The first step included the computational prediction of target genes of DE miRNAs using miRanda (http://www.microrna.org/) and TargetScan (http://www.targetscan.org/) and the selection of targets predicted by both algorithms to use in further analyses. Then, the Pearson’s correlation analysis was performed between the expression of DE miRNAs and their computationally predicted targets detected by RNA-Seq. Significantly correlated miRNA-mRNA pairs (r < −0.5, *P* < 0.05) were selected for further functional analysis. Functional analysis of the miRNA-mRNA pairs revealed that the DE miRNAs were involved in “lymphocyte activation” (miR-133b, miR-146 b, and miR-196 b), “inflammatory response activation” (miR-146 b, miR-184, and miR-1247), “muscle tissue and epithelium development” (miR-146 b and miR-137), as well as “proliferation of endothelial cells” (miR-137, miR-196 b, miR-433, and miR-1247) (Fig. 5C).

## Discussion

It is well established that MAP inhibits macrophage bactericidal activity by blocking phagolysosome formation, a primary effector function of macrophages following intracellular infection^21^. Our analysis reveals that alternative splicing of pre-mRNAs may be a potential mechanism by which MAP evades macrophage killing by altering macrophage maturation and lysosome functions. Especially, *MMD*, a marker gene of macrophages maturation^22^, displayed an increased frequency for an abnormal first exon that could disrupt protein synthesis in the infected tissues, when compared to control tissues. MMD is an ion channel protein and decreased synthesis of this protein may block macrophage maturation^23^. Furthermore, retention of the fourth intron in *ADA* mRNA can produce a truncated protein, resulting in defective lysosome functions^24^. Together, these two splice variants can result in a failure of macrophage maturation and lysosome function, reducing MAP clearance from the site of infection during the early stage of infection. Changes in alternative splicing events following bacterial (*Escherichia coli, in vitro*), viral (*Sendai virus, in vitro*), and parasite (*Crithidia bombi*, in insect) infections have been reported previously, but the impacts of splice variants on protein expression levels are not well studied^18^,^25^,^26^,^27^. Thus, it is necessary to confirm that these two alternative splicing events observed in the present study impact macrophage protein expression levels during MAP infection, *in vitro* and *in vivo*. It is worth to mention that the alternative splicing data presented in this study is preliminary due to the high variations among individuals and lacking of the validation at protein expression level. Besides, RT-qPCR results revealed higher expression of the abnormal isoform of *MMD* (expression of isoform 1/expression of isoform 2 < 1) in both MAP-infected and control samples comparing to those detected by RNA-seq. Although RNA-Seq detected the increase of the abnormal isoform of *MMD* in MAP-infected tissues, it revealed higher expression of normal isoform of *MMD* in both MAP-infected and control samples. Such controversy between RNA-Seq and RT-qPCR may due to the inaccuracy of RNA-Seq in detection of low abundant transcripts, since it has been reported that RNA-Seq cannot accurately detected the abundance of transcripts that have reads number less than 100^28^.

In addition to the influence on macrophages, MAP infection also may have altered toll-like receptor (TLRs) signalling pathway and downstream inflammatory responses by altering miRNAs expression. The expression of miR-146 b, which inhibits the activity of intermediate molecules of the TLR-pathway, such as interleukin-1 receptor-associated kinase 1 (*IRAK1*), interleukin-1 receptor-associated kinase 2 (*IRAK2*) and TNF receptor-associated factor 6 (*TRAF6*)^29^ was significantly increased in infected tissues. The upregulation (fold change = 3.22) of miR-146 b in infected ileum may be one of the mechanisms by which MAP disrupts TLR signalling, following the infection. This is consistent with a previous *in vivo* study that reported inhibition of the TLR signalling pathway at 12 hours after MAP infection in the ileum^30^. Our results suggest that subversion of TLR signalling may be sustained throughout a persistent MAP infection and increased production of miR-146 b may play a key role in this process. There were no significant differences in the expression of TLR signalling molecules (*IRAK1, IRAK2* and *TRAF6*) at the mRNA level, when comparing infected and control compartments. It is still possible, however, that protein expression of these molecules may have been reduced due to post-transcriptional regulation by miR-146 b. Future studies using antibodies to detect TLR signalling molecules (miR-146 b target genes) in MAP-infected tissues may provide in depth understanding on the role of miR-146 b during early infection. Moreover, miR-146 b was negatively correlated with its predicted target genes, interleukin 4-receptor (*IL4R*) (r = −0.67, *P* < 0.01) and spleen tyrosine kinase (*SYK*) (r = −0.59, *P* < 0.01). *SYK* activates the NF-κB-mediated transcription of cytokines^31^,^32^. Their negative correlation with miR-146 b indicates that inflammatory responses triggered via the TLR-signalling pathway may also be inhibited by miR-146 b. These observations suggest that miR-146 b may be able to regulate host responses to MAP infection at multiple stages. Overall, the miRNA results provide further supporting evidences to a previous study reporting the inhibition of TLR-signalling pathway and expression of pro-inflammatory cytokines, following MAP infection^1^.

The RNA-Seq data also suggested that the most relevant function of DE genes in infected ileum was related to “endothelial cell proliferation”. The upregulation of genes, such as cadherin 13 (*CDH13*), semaphorin 6B (*SEMA6B*), dimethylarginine dimethylaminohydrolase 1 (*DDAH1*), vasoactive intestinal peptide (*VIP*) and integrin alpha 1 (*ITGA1*), are consistent with increased proliferation of endothelial cells in the infected tissues^33^,^34^,^35^,^36^,^37^. Activation of endothelial cell proliferation was also suggested by the detected changes in miRNA expression. We identified four DE miRNAs, including miR-1247, miR-137, miR-196 b, and miR-433, with target genes involved in “proliferation of endothelial cells”. For example, increased expression of miR-196 b, an ileum-specific miRNA in young calves^38^, has been reported to increase endothelial cell proliferation in cancer^39^. It is intriguing to consider the possibility that the tissue specificity of this miRNA in the gastrointestinal tract of young calves may contribute to the proclivity for MAP infections to persist in the ileal region of the small intestine. Furthermore, the negative correlation between miR-196 b and secreted protein, acidic, cysteine-rich (*SPARC*) (r = −0.65, *P* < 0.05), an inhibitor of the proliferation of endothelial cells^40^, suggests that upregulation of miR-196 b in infected tissues may increase the proliferation of endothelial cells by inhibiting *SPARC* expression. Proliferation of endothelial cells after MAP infection is one of the causes of granuloma formation^6^, which is a significant feature of JD in ruminants^1^. Granulomas mainly consist of MAP-infected macrophages and provide an organized and protected microenvironment within which MAP can establish a persistent infection in the host^41^. Although, no granulomas were histologically visible at one-month post-infection in our study (data not shown), a previous study reported that granulomas were observed at 3 to 4 weeks after mycobacteria infection^42^. Nevertheless, this study revealed that MAP infection promotes endothelial cell proliferation by changing both mRNA and miRNA expression at the site of infection and these transcriptional changes may support granuloma formation during a persistent infection of MAP.

In the present study, transcriptional changes of immune genes observed at one-month post infection were all related to innate immune response functions but not the induction of adaptive immune responses. This observation is consistent with a previous study that reported the absence of detectable immune responses during the subclinical stage of MAP infection in ileum^43^. The proliferation of regulatory T cells was proposed as a potential mechanism for the suppression of an adaptive immune response^43^. However, similar to Roussey and colleagues^44^, our analysis did not reveal any significant transcriptional changes related to T cell function at one-month post-infection. More detailed studies are required to determine whether there is either the induction of adaptive immune responses in the ileal PPs or recruitment of mucosal effector cells to the site of MAP infection during the subclinical stage of disease.

Pathogen infection leads to host metabolic changes to meet the increasing energy demand associated with a variety of defense responses. Moreover, the pathogenicity of MAP is determined by its capacity to adapt central metabolism to utilize host-derived nutrients^45^. Thus, a persistent MAP infection may alter host metabolism at the site of infection and decrease nutrient absorption by granuloma formation. One of the host metabolic responses during an infection is the increased level of glucose consumption by innate immune cells, such as macrophages and this demand is met by glycogenolysis in muscle tissues, which breakdown stored glycogen into glucose^46^. Macrophages utilize glucose to produce reactive oxygen species when phagolysosome activity is increased for clearance of intracellular pathogens, which can deplete stored glycogen in infected tissues^46^. The transcriptome profiling revealed both a disruption in macrophage functions and a potential disruption of glucose metabolism in the MAP infected ileal compartment. Twenty-one DE genes observed in the present study that were related to glucose metabolism disorders revealed a downregulation in glycogen breakdown into glucose. This suggests a reduction in glycogenolysis or increased glycogen reserves in tissue with MAP infection. To validate these potential changes in host energy metabolism and their link to the MAP infection, we measured glycogen concentrations in MAP infected and control ileal tissues. Glycogen concentrations were increased in infected (0.35 ± 0.10 μmol/g tissue) *vs*. control (0.27 ± 0.044 μmol/g tissue) tissues, but this change was not statistically significant. Moreover, glycogen concentrations in the MAP infected compartments were positively correlated (r = 0.9, *P* = 0.04) with the expression of *ADA*. This observation suggests a possible link between increased glycogen production and genes related to lysosome functions. It will be necessary, however, to follow these changes throughout a longer course of infection to substantiate a causal link between glycogen accumulation and truncated-ADA protein production during MAP infection.

In conclusion, the present study provides insights into new molecular regulatory mechanisms by which MAP may evade host immune responses during the early subclinical stage of infection. We identified DE miRNAs and alternative splicing events that may contribute to host molecular regulatory mechanisms that permit persistent MAP infection during early stage. The surgically isolated intestinal segment model system enabled identification of significant changes between infected and non-infected ileum tissues within the same animal. The overall effect of MAP infection on mRNA expression was limited at one-month post-infection, and DE genes were primarily related to “endothelial cell proliferation”. Furthermore, two genes related to macrophage function (*MMD, ADA*) were confirmed to be differentially spliced in MAP-infected compartments, suggesting a possible mechanism by which MAP escapes the host innate immune response. Profiling miRNA expression revealed that miRNAs might be more responsive than mRNA during the early stage of MAP infection. The integration of DE miRNA with DE mRNAs revealed potential miRNA-mRNA regulatory pairs (miR-196 b and *SPARC*; miR-146 b and *IL4R*; miR-146 b and *SYK*) that were related to MAP-associated changes in “endothelial cell proliferation”, “bacteria recognition”, and “activation of inflammatory responses”. These miRNAs, especially if they are released into the circulation system from infected tissues, may provide potential biomarkers to diagnose subclinical-stage MAP infection. Future studies are needed to further validate the alternative splicing isoform changes at the protein level, to identify the changes in the morphology or number of endothelial cells, and to explore the mechanisms that miRNA regulated mRNA expression in the MAP-infected ileum tissue during the same early infection stage as well as in the later infection stages.

## Materials and Methods

### Animal study and tissue collection

The materials for this study were collected during a previous study^16^ and all experimental protocols were performed following the guidelines approved by the Canadian Council on Animal Care and all procedures were conducted in accordance with the approved protocol. Protocols for animal housing, anesthesia, surgery, MAP infection, and postsurgical care were performed as previously described^16^. Five male, Holstein calves that were 10–14 days old were inoculated with MAP using surgically isolated intestinal segments. Briefly, a 30–35-cm segment of intestine was surgically isolated, proximal to the ileo-cecal fold, and subdivided into three equal compartments using silk ligatures. The distal compartments were injected with 1 − 3 × 10^8^ CFU of MAP strain K10 in a final volume of 5 ml phosphate-buffered saline (PBS). The proximal compartment of the intestinal segment was injected with 5 ml PBS. After surgery, calves were treated with 1.1 mg/kg flunixin (Banamine; Schering Plough Canada Inc., Pointe Claire, Quebec, Canada) for 3 days and with 3 to 4 mg/kg enrofloxacin (Baytril; Bayer Inc.) for 5 days. Calves were fed a whole-milk diet for 4 weeks. One-month post-infection, tissues from the distal (infected) and proximal (control) compartments were collected immediately after euthanizing calves. Each intestinal compartment was opened longitudinally, the contents removed, and longitudinal strips of intestinal tissue measuring 0.5 cm by 3 cm were collected into cryovials, and snap-frozen in liquid nitrogen prior to storage at −80 °C.

### RNA extraction

All samples were processed in a level 2-biosecurity lab at the University of Alberta and tissue samples were ground into a fine powder in a frozen mortar, while immersed in liquid nitrogen. Total RNA was then extracted from 80 mg of tissue powder using mirVana™ miRNA Isolation Kit (Ambion, Carlsbad, CA) following the manufacturer’s instructions. The quality and quantity of the RNA were determined using the Agilent 2100 Bioanalyzer (Agilent Technologies, Santa Clara, CA) and Qubit 2.0 Fluorometer (Invitrogen, Carlsbad, CA), respectively. RNA samples with an integrity number (RIN) higher than 8.0 were used for RNA-Seq library construction.

### RNA-Seq library construction and sequencing

Total RNA (1.0 μg) from each sample was used to construct RNA-Seq libraries using the TruSeq mRNA Sample Preparation kit (Illumina, San Diego, CA) following the manufacturer’s instruction. Individual libraries were then pooled for sequencing according to Illumina’s instruction and sequenced at Génome Québec (Montréal, Canada) using the Illumina HiSeq 2000 system (Illumina). Sequencing was performed as 100 bp paired-end reads. All reads were demultiplexed according to their index sequences with CASAVA version 1.8 (Illumina) and reads that did not pass the Illumina chastity filter were discarded.

### RNA-Seq reads mapping and annotation

RNA-Seq reads were aligned to the bovine genome (UMD 3.1) using Tophat 2.0.11 with default parameters^47^. HTSeq was used (version 0.6.1, http://www-huber.embl.de/users/anders/HTSeq/) to count the number of reads that were mapped to each gene. The expression level of mRNAs in each library was obtained by normalizing reads number to FPM by the following method: FPM = (gene fragments number / total mapped fragments number per library) × 1,000,000. edgeR was used to identify significantly DE mRNAs^19^, and the significances were declared at fold change >1.5 and *P* < 0.05.

### Identification and annotation of alternative splicing events

As suggested by a previous study^48^, 25 million properly paired reads were randomly selected from each RNA-Seq library for further analysis to confirm that the comparison of alternative splicing events was performed without any bias. Tophat 2.0.11 was used to predict the splice junctions. Splicing analysis was performed for events that had at least 20 total RNA-Seq reads^20^. JuncBASE^49^ was used to annotate the entire alternative splicing events (cassette exons, alternative 5′ splice site, alternative 3′ splice site, mutually exclusive exons, coordinate cassette exons, alternative first exons, alternative last exons, and intron retention). Values for Percentage Spliced Index (PSI) were calculated using the formulas provided by a previous study^50^. Fisher’s exact tests were performed on raw read counts from 2 × 2 tables of exclusion and inclusion read counts for each animal to test the different alternative splicing event, as suggested by a previous study^20^. The different alternative splicing events were declared at adjusted *P* < 0.05, and ΔPSI > 10%.

### Construction and analysis of small RNA libraries from ileal tissue

Total RNA (1.0 μ g) from each sample was used to construct a small RNA library using the TruSeq Small RNA Sample Preparation kit (Illumina) and following the manufacturer’s instruction. Individual libraries were then pooled for sequencing according to Illumina’s instruction and sequenced at Génome Québec using the Illumina HiSeq 2000 system (Illumina). Sequencing was performed as 50 bp single reads. All reads were demultiplexed according to their index sequences with CASAVA version 1.8 (Illumina) and reads that did not pass the Illumina chastity filter were discarded.

All small RNA data processing was conducted according to the method described in a previous study^38^. Briefly, sequences with acceptable quality were processed to be short tags by removing the 3′adapter using a Perl script provide by miRDeep2^51^. After trimming the 3′ adaptor sequence, all identical sequences of sizes ranging from 18–25 nt were mapped to the ncRNA sequences (Rfam) to remove non-miRNA small RNA sequences. Then, all sequences were aligned against the corresponding known miRNA precursor sequences (miRBase release version 20) by using the module of quantifier.pl in miRDeep2 with the default parameters to identify known miRNAs. Novel miRNAs were detected using miRDeep2, with the miRDeep2 score cutoff of 5 and more than 20 mapped reads in all samples. The expression of miRNAs in each library was normalized to RPM by the following method: RPM = (miRNA reads number/total mapped reads per library) × 1,000,000. edgeR was used to identify DE miRNAs^19^, and the significances were declared at fold change >1.5 and *P* < 0.05.

### Functional analysis

Ingenuity pathway analysis (IPA, Ingenuity Systems, www.ingenuity.com) was used to identify the functions of DE mRNAs. A threshold of *P* < 0.01 was applied to enrich significant biological functions. The IPA regulation z-score algorithm was used to predict the direction of change for a given function (increase or decrease) according to the fold change of DE genes. A z-score > 0 means that a function is increased, whereas a z-score < 0 indicates a significantly decreased function.

The putative target genes for miRNAs were predicted by miRanda (http://www.microrna.org/) and TargetScan (http://www.targetscan.org/) as described previously^38^. The targets predicted by both algorithms were used for further functional analysis. The target genes of DE miRNAs were uploaded into PANTHER for functional analysis of DE miRNAs^52^. Each analysis was performed using the statistical overrepresentation test option, and significant GO terms were selected at *P* < 0.05 and molecule number >2.

### RT-qPCR validation of differential alternative splicing events

The differential alternative splicing of *MMD* and *ADA* was validated by duplex RT-qPCR using customized primers and probes. Primers and TaqMan^®^ probes (Life Technologies, Carlsbad, CA) were designed based on each event using Custom TaqMan^®^ Assay Design Tool (https://www.thermofisher.com/). The primers and probes are presented as followed (Supplementary Table S5):

Isoform 1 of *MMD*: Forward primer: 5′-AATGGCCGCTACAAGCCAAC-3′; Reverse primer: 5′- CATCAGAGAGCCGGTGAAGG-3′; Probe: 5′FAM-AATGGCCGGAACAATGAGGAATGC-NFQ-MGB3′;

Isoform 2 of *MMD*: Forward primer: 5′-CAGTGCTGATCCTATCTGGAAGA-3′; Reverse primer: 5′-CATCAGAGAGCCGGTGAAGG-3′; Probe: 5′VIC-AGTTTGCATTCTTTCCTCATTGTTCCGG-NFQ-MGB3′;

Isoform 1 of *ADA*: Forward primer: 5′-TGTGGAGATGAAGGCCAAGG-3′; Reverse primer: 5′-CCAGTGACACCACCTCATCC-3′; Probe: 5′FAM-AGCCGATCCCCTGG AACCAGGCTGAAGGGG-NFQ-MGB3′;

Isoform 2 of *ADA*: Forward primer: 5′-CTCCTTCCTCTCTCTCCTACC-3′; Reverse primer: 5′-GATGAGGTGGTGTCACTGG; Probe: 5′VIC-TTCCCCACACACAGAGGGGACCTCACCCCG-NFQ-MGB3′.

Total RNA (1.0 μ g) from each sample was treated with DNAase I (Invitrogen), and reverse-transcribed to cDNA was performed using SuperScript II reverse transcriptase following the manufacturer’s protocol (Invitrogen). A total of 100 ng cDNA, 10.0 ptase fol^®^ Fast Advanced Master Mix, primers (final concentrations was 900 nM) and probe (final concentration was 250 nM) were used in a 20 μl reaction, which was suggested by TaqMan^®^ Gene Expression Assays Protocol. Primers and probes for the two isoforms of *MMD* or *ADA* were added to one reaction. The fluorescence signals were detected with StepOnePlus Real-Time PCR System (Life Technologies) with the following cycling times and temperatures: 50 °C for 2 mins, 95 °C for 20 s and 40 cycles of 94 °C for 1 s, 60 °C for 20 s. The cycle threshold (Ct) values for the isoforms in different samples have been listed in Supplementary Table S6. The alternative splicing events were detected by evaluating the ratio between the expressions of the two isoforms: Expression _isoform 1_/Expression _isoform 2_ = 2^(Ct isoform 2 -Ct Expression even)^.

### Experimental validation of miRNA expression using stem-loop RT-qPCR

Expression of 16 DE miRNAs identified by RNA-Seq was validated using stem-loop RT-qPCR with TAQMAN miRNA assays (Applied Biosystems). Briefly, cDNAs were reverse-transcribed from 10 ng of total RNA using specific miRNA RT primer (Applied Biosystems) and then were amplified using a TAQMAN miRNA assay. Fluorescence signal was detected with StepOnePlus™ Real-Time PCR System (Applied Biosystems). U6 snRNA (Applied Biosystems) was used as an internal control and the relative expression of miRNA was calculated by ΔΔCt method. Students’ t-test was used to compare miRNA expression between infected and control tissues. Differences were considered statistically different at *P* < 0.05 and analyses were performed in R using t.test function.

### Glycogen concentration measurement in ileal tissue

Glycogen concentration was measured using Glycogen Assay Kit (Cayman Chemical, Ann Arbor, MI), according to the manufacturer’s instructions. Briefly, the frozen tissues were minced into small pieces, and then homogenized to disrupt cells. Homogenate was centrifuged and 50 μl of supernatant was used for glycogen measurement. The reagents of Glycogen Assay Kit (Cayman Chemical) were able to hydrolyze glycogen and generate highly fluorescent product resorufin (Glycogen Assay Kit Manual). Fluorescence (Ex/Em = 535/587 nm) was measured with the SpectraMax M3 system (Molecular Devices, Sunnyvale, CA).

### Data submission

The raw sequencing data including putative novel miRNA information have been deposited at publicly available NCBI’s Gene Expression Omnibus Database (http://www.ncbi.nlm.nih.gov/geo/). The data are accessible through GEO Series accession number GSE74394 (http://www.ncbi.nlm.nih.gov/geo/query/acc.cgi?acc=GSE74394).

## Additional Information

**How to cite this article**: Liang, G. *et al*. Altered microRNA expression and pre-mRNA splicing events reveal new mechanisms associated with early stage *Mycobacterium avium* subspecies *paratuberculosis* infection. *Sci. Rep.* **6**, 24964; doi: 10.1038/srep24964 (2016).

## Supplementary Material

Supplementary Information

## Figures and Tables

**Table 1:** The differentially expressed genes between MAP-infected and control tissues detected by RNA-Seq.

Gene Symbol	Fold change	Regulation	Gene Symbol	Fold change	Regulation	Gene Symbol	Fold change	Regulation
PNMA6A	0.27	D	ACE2	1.63	U	ABCC2	1.82	U
DEFB	0.40	D	KIFC3	1.64	U	SPSB4	1.82	U
EIF1AY	0.46	D	EFNA5	1.65	U	ARHGEF4	1.87	U
RPL36A	0.48	D	FAM46B	1.66	U	SEMA6B	1.88	U
SAA3	0.52	D	RHOBTB3	1.67	U	PDK4	1.89	U
Bta-mir-425	0.55	D	MB21D2	1.67	U	ENPP3	1.90	U
CFAP46	0.55	D	COL4A1	1.68	U	PCK1	1.92	U
BEST2	0.56	D	VIP	1.70	U	G3 × 7I8	1.94	U
GPR113	0.57	D	TINAGL1	1.70	U	FRMD3	1.95	U
PTPRO	0.58	D	DNAJA4	1.70	U	CDH13	1.97	U
Bta-mir-421	0.59	D	DDAH1	1.70	U	ATP8B1	1.97	U
FCER1A	0.61	D	WFS1	1.72	U	PNCK	1.97	U
MPP3	0.62	D	KLK10	1.72	U	TMEM132C	1.99	U
MYBPH	0.65	D	IYD	1.73	U	ZCCHC12	2.01	U
MAFF	1.52	U	TRIM40	1.73	U	PABPC5	2.01	U
RND3	1.52	U	CAV2	1.73	U	TRPM6	2.02	U
ERI1	1.52	U	ME3	1.74	U	IGFBP2	2.03	U
SYT4	1.55	U	CPQ	1.74	U	HSPA6	2.03	U
PECI	1.55	U	HOXD9	1.75	U	SAMD4A	2.06	U
NOS3	1.57	U	DUSP26	1.75	U	LOC101906113	2.10	U
MLF1	1.57	U	TMEM56	1.76	U	DPF3	2.15	U
ART5	1.58	U	AGPAT9	1.76	U	LEKR1	2.64	U
COL4A2	1.58	U	E1BLE0	1.78	U	PDE7B	2.65	U
ITGA1	1.59	U	EXOC3L2	1.79	U	GRP	2.84	U
ADSSL1	1.62	U	CCDC70	1.79	U	DYTN	3.36	U
PRSS23	1.62	U	ANGPTL4	1.82	U	PAK7	4.41	U
SLC16A13	1.63	U	GJB3	1.82	U	PSAPL1	7.03	U

Notes: “D” indicates downregulated genes in MAP-infected *vs*. control tissues; “U” indicates upregulated genes in MAP-infected *vs*. control tissues; Fold changes were defined as ratios of arithmetic means of FPM when compare MAP-infected *vs*. control tissues. The significances were declared at fold change > 1.5 and *P* < 0.05 (edgeR analysis).
