# Supplementary material for: Altered microRNA expression and pre-mRNA splicing events reveal new mechanisms associated with early stage Mycobacterium avium subspecies paratuberculosis infection
Source: Sci Rep. 2016 Apr 22;6:24964. doi: 10.1038/srep24964 (PMC4840452; doi:10.1038/srep24964)
Supplement: Supplementary Information [file srep24964-s1.pdf]

Supplementary Information

Altered microRNA expression and pre-mRNA splicing events reveal new mechanisms associated with early stage *Mycobacterium avium* subspecies *paratuberculosis* infection

Guanxiang Liang[1], Nilusha Malmuthuge[1], Yongjuan Guan[2], Yuwei Ren[1, 3], Philip J. Griebel[4, 5], Le Luo Guan[1*]

[1]Department of Agricultural, Food and Nutritional Science, University of Alberta, Edmonton, AB, Canada; [2]UWA Institute of Agriculture and School of Animal Biology, University of Western Australia, Crawley, WA, Australia; [3]Key Laboratory of Agricultural Animal Genetics, Breeding and Reproduction of Ministry of Education, Huazhong Agriculture University, Wuhan, Hubei, China; [4]Vaccine and Infectious Disease Organization, University of Saskatchewan, Saskatoon, SK, Canada; [5]School of Public Health, University of Saskatchewan, Saskatoon, SK, Canada

*Corresponding author, 416F Agriculture/Forestry Centre, University of Alberta, Edmonton, AB, Canada, T6G 2P5, Tel.: +1(780) 4922480. Fax: +1(780) 49244265, E-mail: lguan@ualberta.ca

Supplementary Table S1:

The summary of RNA-Seq data.

	ID	Reads	Map ratio to genome (reads)	Gene # (FPM > 1)	Small RNA reads	Reads mapped to miRNA database	miRNA # (RPM > 1)
**Control**	C1	14840180	90.10%	14520	1830610	1442499	377
	C2	17980237	89.80%	14316	901881	712290	411
	C3	16235901	90.30%	14192	1885526	1376780	387
	C4	17904466	90.20%	14527	1628604	1280173	357
	C5	16959044	90.30%	14663	3226354	2760456	345
**Infected**	M1	18839407	90.40%	14501	1411091	1048177	363
	M2	16739948	90.00%	14372	980787	722819	405
	M3	22545529	90.00%	14304	3172164	2516249	366
	M4	16150699	89.70%	14478	1685827	1418709	345
	M5	15618095	90.00%	14493	1066491	791962	397

Supplementary Table S2:

The GO term enrichment of 3000 most highly expressed genes.

Category	Term	Count	%	PValue
GOTERM_BP_ALL	GO:0009987~cellular process	1255	43.29079	5.60E-70
GOTERM_BP_ALL	GO:0044237~cellular metabolic process	828	28.56157	9.34E-44
GOTERM_BP_ALL	GO:0044237~cellular metabolic process	828	28.56157	9.34E-44
GOTERM_BP_ALL	GO:0044238~primary metabolic process	848	29.25147	4.68E-34
GOTERM_BP_ALL	GO:0044238~primary metabolic process	848	29.25147	4.68E-34
GOTERM_BP_ALL	GO:0044260~cellular macromolecule metabolic process	609	21.00724	3.63E-30
GOTERM_BP_ALL	GO:0008152~metabolic process	951	32.80442	1.85E-27
GOTERM_BP_ALL	GO:0043170~macromolecule metabolic process	674	23.2494	5.83E-26
GOTERM_BP_ALL	GO:0010467~gene expression	313	10.79683	3.55E-25
GOTERM_BP_ALL	GO:0006412~translation	121	4.173853	5.02E-24
GOTERM_BP_ALL	GO:0044267~cellular protein metabolic process	382	13.17696	1.15E-21
GOTERM_BP_ALL	GO:0006139~nucleobase, nucleoside, nucleotide and nucleic acid metabolic process	339	11.69369	2.09E-20
GOTERM_BP_ALL	GO:0016043~cellular component organization	279	9.624008	8.16E-19
GOTERM_BP_ALL	GO:0034641~cellular nitrogen compound metabolic process	365	12.59055	1.43E-18
GOTERM_BP_ALL	GO:0019538~protein metabolic process	446	15.38462	8.86E-18
GOTERM_BP_ALL	GO:0044249~cellular biosynthetic process	355	12.2456	2.14E-17
GOTERM_BP_ALL	GO:0006807~nitrogen compound metabolic process	370	12.76302	3.82E-17
GOTERM_BP_ALL	GO:0016071~mRNA metabolic process	71	2.44912	5.71E-16
GOTERM_BP_ALL	GO:0009058~biosynthetic process	365	12.59055	1.53E-15
GOTERM_BP_ALL	GO:0016070~RNA metabolic process	136	4.691273	6.11E-15
GOTERM_BP_ALL	GO:0006397~mRNA processing	64	2.207658	6.52E-15
GOTERM_BP_ALL	GO:0009059~macromolecule biosynthetic process	261	9.003105	7.67E-15
GOTERM_BP_ALL	GO:0034645~cellular macromolecule biosynthetic process	259	8.934115	1.07E-14
GOTERM_BP_ALL	GO:0008380~RNA splicing	50	1.724733	6.24E-13
GOTERM_BP_ALL	GO:0044248~cellular catabolic process	137	4.725768	2.23E-12
GOTERM_BP_ALL	GO:0009056~catabolic process	171	5.898586	2.89E-12
GOTERM_BP_ALL	GO:0033036~macromolecule localization	157	5.415661	5.69E-12
GOTERM_BP_ALL	GO:0046907~intracellular transport	96	3.311487	7.72E-12
GOTERM_BP_ALL	GO:0051641~cellular localization	123	4.242842	1.92E-11

Supplementary Table S3:

The alternative splicing events information.

Symbol	as_event_type	chr	strand	exclusion_junctions	inclusion_junctions	exclusion_exons	inclusion_exons	intron-exon_junctions
ADA	intron_retention	chr13	-	chr13:73756425-73757159				chr13:73756424-73756425;chr13:73757159-73757160
AP3D1	intron_retention	chr7	+	chr7:22732097-22732473				chr7:22732096-22732097;chr7:22732473-22732474
ASAH1	intron_retention	chr27	+	chr27:18302473-18306297				chr27:18302472-18302473;chr27:18306297-18306298
C4BPA	alternative_acceptor	chr16	+	chr16:66041590-66043301	chr16:66041590-66043239		chr16:66043240-66043301	chr16:66043301-66043302
CHUK	alternative_acceptor	chr26	-	chr26:20968047-20970266	chr26:20969397-20970266		chr26:20968047-20969396	chr26:20968046-20968047
CIITA	intron_retention	chr25	+	chr25:9661059-9662075				chr25:9661058-9661059;chr25:9662075-9662076
CLN3	cassette	chr25	-	chr25:26305441-26306857	chr25:26305441-26305519;chr25:26305567-26306857		chr25:26305520-26305566	
FCRL1	alternative_acceptor	chr3	+	chr3:12642006-12642344;chr3:12642006-12642319	chr3:12642006-12642303		chr3:12642304-12642319;chr3:12642320-12642344	chr3:12642319-12642320;chr3:12642344-12642345
GBA	intron_retention	chr3	+	chr3:15462302-15462986				chr3:15462301-15462302;chr3:15462986-15462987
IFI30	alternative_donor	chr7	-	chr7:4983392-4984416;chr7:4983392-4986093;chr7:4983392-4983886;chr7:4983392-4984769	chr7:4983392-4983640		chr7:4983641-4983886;chr7:4983887-4984416;chr7:4984417-4984769;chr7:4984770-4986093	chr7:4983886-4983887;chr7:4984416-4984417;chr7:4984769-4984770;chr7:4986093-4986094
LY75	intron_retention	chr2	+	chr2:36608555-36609489				chr2:36608554-36608555;chr2:36609489-36609490
MMD	alternative_first_exon	chr19	-	chr19:5834814-5835370,chr19:5834814-5839687;chr19:5834814-5839691	chr19:5834814-5835733	chr19:5839692-5839782	chr19:5835734-5836144	
NFKBIZ	intron_retention	chr1	+	chr1:46549511-46549737				chr1:46549510-46549511;chr1:46549737-46549738
RFXANK	intron_retention	chr7	-	chr7:3987874-3988232				chr7:3987873-3987874;chr7:3988232-3988233
RIPK2	alternative_acceptor	chr14	-	chr14:76234350-76235385	chr14:76235301-76235385		chr14:76234350-76235300	chr14:76234349-76234350
VPS16	intron_retention	chr13	-	chr13:52786177-52786276				chr13:52786176-52786177;chr13:52786276-52786277

Supplementary Table S4:

Protein sequence analysis of alternative splicing events.

Protein sequence of Isoform 1 for *MMD*:

Met N H R A P A N G R Y K P T C Y E H A A N C Y T H A F L I V P A I V G S A L L H R L

S D D C W E K I T A W I Y G Met G L C A L F I V S T V F H I V A W K K S H L R T V E H

W F H Met C D R Met V I Y F F I A A S Y A P W L N L R E L G P L A S H Met R W F I W L

Met A A G G T I Y V F L Y H E K Y K V I E L F F Y L T Met G F S P A L V V T S Met N N

T D G L H E L A C G G L I Y C L G V V F F K S D G I I P F A H A I W H L F V A T A A A

V H Y Y A I W K Y L Y R S P T D F Met R H L Stop

Protein sequence of Isoform 2 for *MMD*:

G T L I Stop

Protein sequence of Isoform 1 for *ADA*:

Met A Q T P A F N K P K V E L H V H L D G A I K P E T I L Y Y G R K R G I A L P A D T P

E E L Q N I I G Met D K P L S L P E F L A K F D Y Y Met P A I A G C R E A V K R I A Y E

F V E Met K A K D G V V Y V E V R Y S P H L L A N S K V E P I P W N Q A E G D L T P

D E V V S L V N Q G L Q E G E R D F G V K V R S I L C C Met R H Q P S W S S E V V E L

C K K Y R E Q T V V A I D L A G D E T I E G S S L F P G H V Q A Y A E A V K S G V H R

T V H A G E V G S A N V V K E A V D T L K T E R L G H G Y H T L E D T T L Y N R L R

Q E N Met H F E V C P W S S Y L T G A W K P D T E H P V V R F K N D Q V N Y S L N T

D D P L I F K S T L D T D Y Q Met T K N E Met G F T E E E F K R L N I N A A K S S F L P

E D E K K E L L D L L Y K A Y G Met P S P A S A E Q C L Stop

Protein sequence of Isoform 2 for *ADA*:

Met A Q T P A F N K P K V E L H V H L D G A I K P E T I L Y Y G R K R G I A L P A D T P

E E L Q N I I G Met D K P L S L P E F L A K F D Y Y Met P A I A G C R E A V K R I A Y E

F V E Met K A K D G V V Y V E V R Y S P H L L A N S K V E P I P W N Q A E Stop

Supplementary Table S5:

The primers of RT-qPCR validation for alternative splicing events.

MMD mRNA:

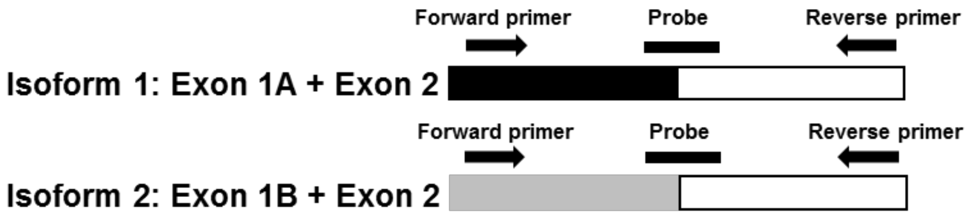

Isoform 1 of *MMD*: Forward primer: 5´-AATGGCCGCTACAAGCCAAC-3´; Reverse primer: 5´- CATCAGAGAGCCGGTGAAGG-3´; Probe: 5´FAM-AATGGCCGGAACAATGAGGAATGC-NFQ-MGB3´;

Isoform 2 of *MMD*: Forward primer: 5´-CAGTGCTGATCCTATCTGGAAGA-3´; Reverse primer: 5´-CATCAGAGAGCCGGTGAAGG-3´; Probe: 5´VIC-AGTTTGCATTCTTTCCTCATTGTTCCGG-NFQ-MGB3´;

ADA mRNA:

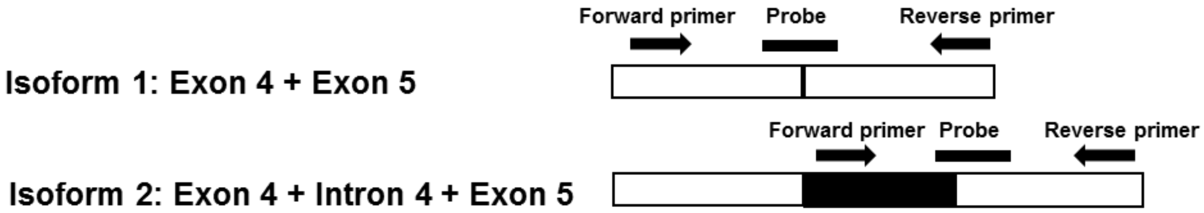

Isoform 1 of *ADA*: Forward primer: 5´-TGTGGAGATGAAGGCCAAGG-3´; Reverse primer: 5´-CCAGTGACACCACCTCATCC-3´; Probe: 5´FAM-AGCCGATCCCCTGGAACCAGGCTGAAGGGG-NFQ-MGB3´;

Isoform 2 of *ADA*: Forward primer: 5´-CTCCTTCCTCTCTCTCCTACC-3´; Reverse primer: 5´-GATGAGGTGGTGTCACTGG; Probe: 5´VIC-TTCCCCACACACAGAGGGGACCTCACCCCG-NFQ-MGB3´.

Supplementary Table S6:

The cycle threshold (Ct) values for differnt isoforms in each sample

	MMD		ADA	
	**Isoform 1**	**Isoform 2**	**Isoform 1**	**Isoform 2**
**C1**	28.29	25.97	25.55	28.43
**M1**	29.3	26.39	27.29	28.01
**C2**	28.81	26.97	25.32	28.92
**M2**	28.83	26.18	25.84	26.90
**C3**	29.88	27.27	24.51	27.92
**M3**	29.75	25.34	25.70	25.75
**C4**	33.86	31.4	26.28	27.87
**M4**	32.81	26.02	26.00	26.19
